# Influence of monolayer, spheroid, and tumor growth conditions on chromosome 3 gene expression in tumorigenic epithelial ovarian cancer cell lines

**DOI:** 10.1186/1755-8794-1-34

**Published:** 2008-08-07

**Authors:** Neal AL Cody, Magdalena Zietarska, Ali Filali-Mouhim, Diane M Provencher, Anne-Marie Mes-Masson, Patricia N Tonin

**Affiliations:** 1 Department of Human Genetics, McGill University, Montreal, Canada; 2 Centre de Recherche du Centre Hospitalier de l'Université de Montréal (CHUM), Institut du cancer de Montréal, Montreal, Canada; 3 Division of Gynecologic Oncology, Université de Montréal, Montreal, Canada; 4 Département de médicine, Université de Montréal, Montreal, Canada; 5 The Research Institute of the McGill University Health Centre, and Department of Medicine, McGill University, Montreal, Canada

## Abstract

**Background:**

Expression microarray analyses of epithelial ovarian cancer (EOC) cell lines may be exploited to elucidate genetic and epigenetic events important in this disease. A possible variable is the influence of growth conditions on discerning candidates. The present study examined the influence of growth conditions on the expression of chromosome 3 genes in the tumorigenic EOC cell lines, OV-90, TOV-21G and TOV-112D using Affymetrix GeneChip^® ^HG-U133A expression microarray analysis.

**Methods:**

Chromosome 3 gene expression profiles (n = 1147 probe sets, representing 735 genes) were extracted from U133A expression microarray analyses of the EOC cell lines OV-90, TOV-21G and TOV-112D that were grown as monolayers, spheroids or nude mouse xenografts and monolayers derived from these tumors. Hierarchical cluster analysis was performed to compare chromosome 3 transcriptome patterns of each growth condition. Differentially expressed genes were identified and characterized by two-way comparative analyses of fold-differences in gene expression between monolayer cultures and each of the other growth conditions, and between the maximum and minimum values of expression of all growth conditions for each EOC cell line.

**Results:**

An overall high degree of similarity (> 90%) in gene expression was observed when expression values of alternative growth conditions were compared within each EOC cell line group. Two-way comparative analysis of each EOC cell line grown in an alternative condition relative to the monolayer culture showed that overall less than 15% of probe sets exhibited at least a 3-fold difference in expression profile. Less than 23% of probe sets exhibited greater than 3-fold differences in gene expression in comparisons of the maximum and minimum value of expression of all growth conditions within each EOC cell line group. The majority of these differences were less than 5-fold. There were 17 genes in common which were differentially expressed in all EOC cell lines. However, the patterns of expression of these genes were not necessarily the same for each growth condition when one cell line was compared with another.

**Conclusion:**

The various alternative *in vivo *and *in vitro *growth conditions of tumorigenic EOC cell lines appeared to modestly influence the global chromosome 3 transcriptome supporting the notion that the *in vitro *cell line models are a viable option for testing gene candidates.

## Background

The molecular genetic analysis of ovarian cancer has been facilitated by the establishment and characterization of spontaneously immortalized epithelial ovarian cancer (EOC) cell lines that have been derived from malignant cells by long-term growth in cell culture [1]. In our laboratory, we have studied the properties of three EOC cell lines derived from malignant ovarian tumors (TOV-21G and TOV112D) and ascites (OV-90) [2,3]. These EOC cell lines were derived from patient samples prior to chemotherapy. They have been extensively characterized and shown to exhibit many of molecular genetic features, cytogenetic anomalies, and somatic mutations in tumor suppressor genes frequently associated with malignant ovarian cancers [2-4]. An attractive feature of these EOC cell lines is that they develop tumors at subcutaneous and intraperitoneal sites in nude mouse xenograft models [2]. The phenotypes of the EOC cell lines are also reflected in global analyses of gene expression using large-scale gene expression microarrays analyses where the differentially expressed genes have been shown to overlap with those observed independently in the molecular analyses of ovarian cancers [5-11]. The application of various growth conditions to capture the full spectrum of the disease along with large-scale gene expression analyses could be important in our understanding of the biological and genetic factors that influence the phenotypic characteristics of the disease [1,12].

A possible variable in the application of EOC cell line models is the influence of growth conditions on discerning and then characterizing gene candidates which initially exhibit differential gene expression in *in vitro *EOC cell line models. Recently, our group has reported on global differences in gene expression between EOC cell lines that were cultured as monolayers, spheroids, or nude mouse xenografts suggesting that microenviroment could impact the transcriptome [13]. To further assess the variability of gene expression of EOC cell lines propagated in different contexts, we have extracted chromosome 3 gene expression profiles from the Affymetrix expression microarray data from three tumorigenic EOC cell lines, TOV-21G, TOV-112D and OV-90, that have been propagated as monolayers, spheroids or nude mouse xenografts, and monolayers derived from these xenografts [13]. We have focused our analysis on the chromosome 3 gene expression because of our interest in elucidating genes located on this chromosome in ovarian cancer and the use of these well established EOC cell lines as models to both identify and characterize chromosome 3 gene candidates potentially important in this disease [7,8,14,15]. These EOC cell lines were derived from ovarian cancer samples from chemotherapy naïve patients and have been shown to exhibit unique karyotypic abnormalities [2]. Both OV-90 and TOV-112D exhibit complex karyotypic anomalies consistent with those typically seen in the majority of EOCs, whereas TOV-21G exhibited an atypical diploid karyotype with trisomy 10 as the only gross abnormality [2,16]. Karyotype analysis has demonstrated evidence of an unique chromosome 3 abnormality in OV-90 comprised of a chromosome 22 derived homogeneously staining region replacing the 3p arm but not affecting the 3q arm [2,5]. In particular, OV-90 has emerged as an interesting *in vitro *model with the potential for identifying and testing chromosome 3 tumor suppressor genes because of extensive loss of heterozygosity of the 3p arm [15], and the recent demonstration of suppression of tumorigenicity in chromosome 3 fragment transfer experiments attributable to functional complementation of 3p genes [14]. The EOC cell line TOV-21G has shown no evidence of chromosome 3 karyotypic abnormalities [2] but has demonstrated evidence of microsatellite instability consistent with mismatch repair anomalies [3].

The present study was focused on addressing the magnitude and extent of transcriptome modifications for different EOC cell line models that may be influenced by tumor microenvironment. As each cell line exhibits unique molecular genetic characteristics comparisons of chromosome 3 transcriptome modifications were made with respect to gene expression profiles generated with each growth condition within each experimental cell line model.

## Methods

### EOC cell lines

The EOC cell lines were derived from a stage III/grade 3 clear cell carcinoma (TOV-21G), a stage IIIc/grade 3 endometrioid carcinoma (TOV-112D), and from the ascites fluid of a stage IIIc/grade 3 adenocarcinoma (OV-90), all from chemotherapy naïve patients, as described [2]. Cells were cultured in OSE medium consisting of 50:50 medium 199:105 (Sigma), with 2.5 μg/mL amphotericin B and 50 μg/mL gentamicin [2]. Culture media was supplemented with 10% FBS.

### Source of chromosome 3 expression profiles

Chromosome 3 gene expression profiles were extracted from normalized Affymetrix GeneChip^® ^HG-U133A microarray analyses of the OV-90, TOV-21G and TOV-112D EOC cell lines that were each grown under different conditions as described previously [13], and will be made available at Gene Expression Omnibus . These conditions include monolayer cultures (L), spheroid cultures (S), *nude *mouse xenografts at subcutaneous (TSC) or intraperitoneal (TIP) sites, and monolayer cultures of subcutaneous (LSC) and intraperitoneal (LIP) xenografts, as described previously [13]. Data normalization, which is intended to eliminate systematic biases when comparing expression values from independently derived GeneChip^® ^experiments, was achieved from the raw expression data using the MAS5 software (Affymetrix Microarray Suite^®^) by multiplying the value for an individual probe set by 100 and dividing by the mean of the raw expression values for the given sample data set as described previously [5,10,17]. The software also generates a reliability score for each probe set, which reflects the level of non-specific binding. A high reliability score of Present (P call) represents minimal hybridization to the mismatch probe set and consistent hybridization across all matched probes, in contrast to a borderline score of Marginal (M call) or a score of Ambiguous (A call).

The normalized data set was then used to extract the expression profiles associated with probe sets representing chromosome 3 genes. Probe sets corresponding to chromosome 3 genes were identified using the Affymetrix NetAffx Batch Query tool  and the UniGene *Homo sapiens *database, based on UniGene Build 198 . Additional mapping information was obtained from the University of California Santa Cruz (UCSC) Human Genome Browser database, March 2006 (NCBI Build 36.1) hg 18 assembly . Based on these databases, 1147 probe sets were identified that mapped to chromosome 3, representing 735 genes and ESTs. Chromosome 3 alignment of probe sets (represented genes) was determined based on the UCSC Human Genome Browser database, where 535 probe sets mapped to genes on the chromosome 3p arm and 612 probe sets mapped to genes on the chromosome 3q arm.

The normalized expression data sets were also rescaled to eliminate systematic biases due to low expression values. Low values with A-calls are considered to be technical noise, which may influence fold-difference comparisons and overestimate expression differences that result from high variability of low expression values. Probe sets containing A-calls may also reflect either absent expression or poorly designed probe sets [5]. To reduce this technical noise, values below 15 were reassigned a threshold value of 15, based on the mean expression value of data with low reliability scores of the chromosome 3 extracted probe set data.

### Hierarchical Cluster Analysis

Hierarchical cluster analysis was performed on normalized and rescaled gene expression data analyzed using Bioconductor, an open-source software library for the analyses of genomic data [18] based on R, a language and environment for statistical computing and graphics . In order to determine the significance of the differential expression, modified t-tests were performed with Bioconductor's *limma *package, where p-values from the resulting comparison were adjusted for multiple testing as described [19]. This method controls for the false discovery rate, which was set to 0.05. Bioconductor's *genefilter *package was used to filter out probe sets with insufficient variation in gene expression across all tested samples for the analysis of each EOC cell line data set. In the remaining expression values, a log base 2 scale of at least 0.5 for the interquartile range was required across all tested samples for each EOC cell line group as described. Hierarchical clustering analysis was performed with R's *cluster *package, using the Pearson correlation distance.

### Two-way comparative analyses

Two-way comparative analyses based on fold differences of expression values were performed on normalized and rescaled gene expression data derived from each EOC cell line. The expression values with at least one high-reliability score or P call for each EOC cell line sample set (data containing expression values generated from each growth condition) were evaluated in two-way comparative analyses. Differentially expressed genes were defined as those which exhibited at least a 3-fold difference in two-way comparative analyses with expression value for monolayer culture and each growth condition, or between the maximum and minimum value of expression observed within a set of data for each EOC cell line.

## Results

### Hierarchical cluster analysis

**Figure 1:**
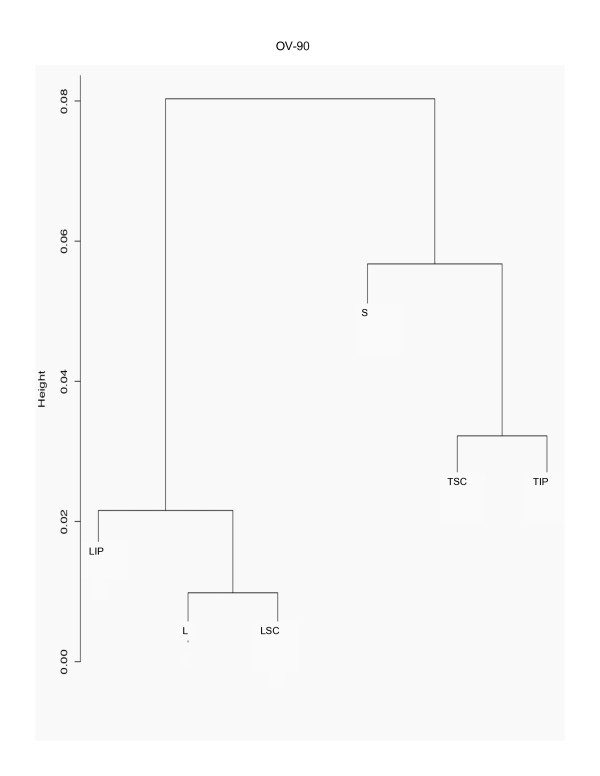
**Hierarchical cluster analysis of OV-90 grown in different conditions**. Hierarchical clustering of normalized chromosome 3 gene expression data sets derived from OV-90 grown as monolayer culture (L), and the alternative growth conditions consisting of spheroid cultures (S), tumors derived from xenograft tumors from subcutaneous (TSC) or intraperitoneal (TIP) injection sites in nude mice, and monolayer cultures derived from these tumors (LSC and LIP). The analysis was carried out using R's cluster package with the Pearson correlation distance where the y-axis 'height' represents the 1 minus the correlation distance. Only part of the clustering analysis is shown which includes the distal branches where the highest degree of correlation begins to deviate for each growth condition (at around 92%).

**Figure 2:**
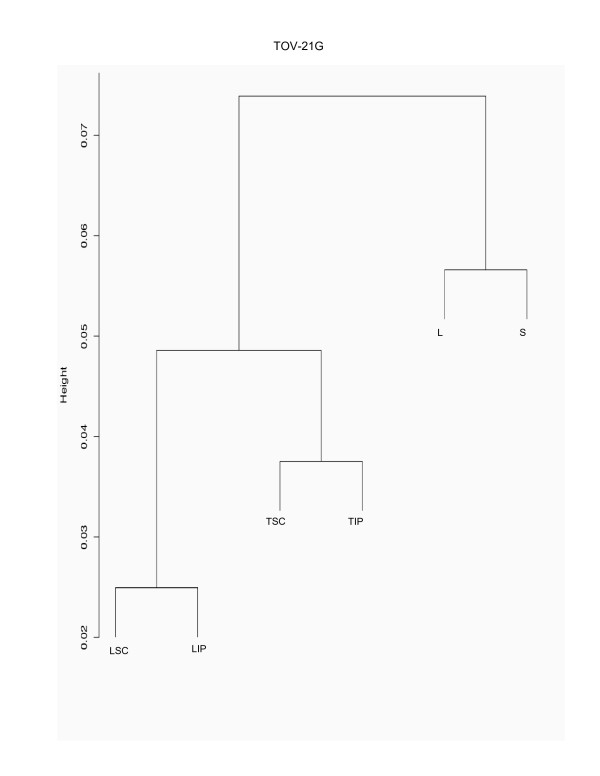
**Hierarchical cluster analysis of TOV-21G grown in different conditions**. Hierarchical clustering of normalized chromosome 3 gene expression data sets derived from TOV-21G grown as monolayer culture (L), and the alternative growth conditions consisting of spheroid cultures (S), tumors derived from xenograft tumors from subcutaneous (TSC) or intraperitoneal (TIP) injection sites in nude mice, and monolayer cultures derived from these tumors (LSC and LIP). The analysis was carried out using R's cluster package with the Pearson correlation distance where the y-axis 'height' represents the 1 minus the correlation distance. Only part of the clustering analysis is shown which includes the distal branches where the highest degree of correlation begins to deviate for each growth condition (at around 93%).

**Figure 3:**
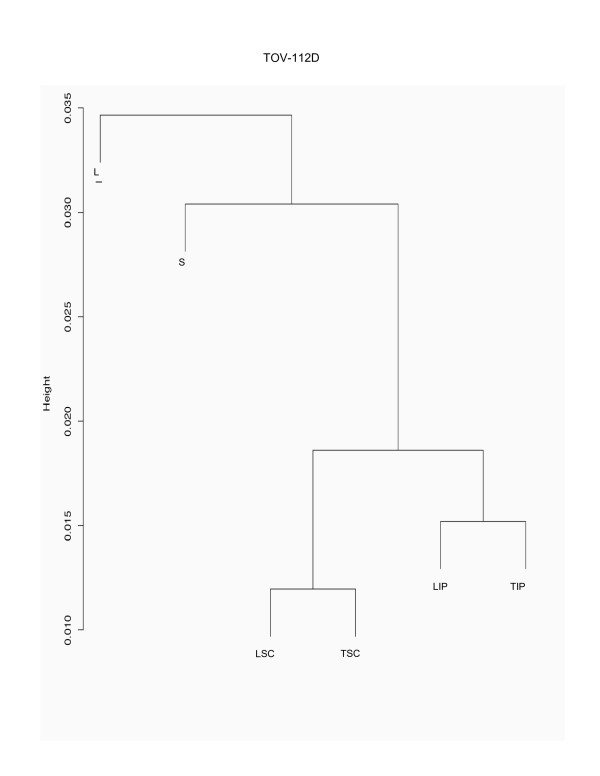
**Hierarchical cluster analysis of TOV-112D grown in different conditions**. Hierarchical clustering of normalized chromosome 3 gene expression data sets derived from TOV-112D grown as monolayer culture (L), and the alternative growth conditions consisting of spheroid cultures (S), tumors derived from xenograft tumors from subcutaneous (TSC) or intraperitoneal (TIP) injection sites in nude mice, and monolayer cultures derived from these tumors (LSC and LIP). The analysis was carried out using R's cluster package with the Pearson correlation distance where the y-axis 'height' represents the 1 minus the correlation distance. Only part of the clustering analysis is shown which includes the distal branches where the highest degree of correlation begins to deviate for each growth condition (at around 96.5%).

Hierarchical cluster analysis of chromosome 3 gene expression data from each EOC cell line grown in monolayer cultures (L) and alternative growth conditions such as spheroid cultures (S), nude mouse xenografts at subcutaneous (TSC) or intraperitoneal (TIP) sites, and monolayer cultures of subcutaneous (LSC) and intraperitoneal (LIP) xenografts is shown in Figures 1, 2 and 3. When looking at the major branches, the xenograft-derived monolayer cultures (LSC and LIP) cluster with the xenografts (TSC and TIP) themselves in the case of TOV-21G (Figure 2) and TOV-112D (Figure 3). In contrast, OV-90 shows two major branches separating the spheroid (S) and xenografts (TSC and TIP) from all monolayer cultures (L, LSC and LIP) (Figure 1). It should be noted that a combined hierarchical cluster analysis of all EOC cell line data sets also results in a similar branching pattern where each EOC cell line clusters within its own grouping rather than with culture condition (data not shown) and this result is consistent with the hierarchical cluster analysis of whole genome transcriptome of these EOC cell line propagated in different contexts [13]. However, an overall high degree of correlation (> 90%) in gene expression was observed when expression values of different growth conditions were compared within each EOC cell line group, suggesting a limited number of chromosome 3 probe sets have altered expression profiles due to the growth conditions.

### Two-way comparisons relative to the reference monolayer culture

**Figure 4:**
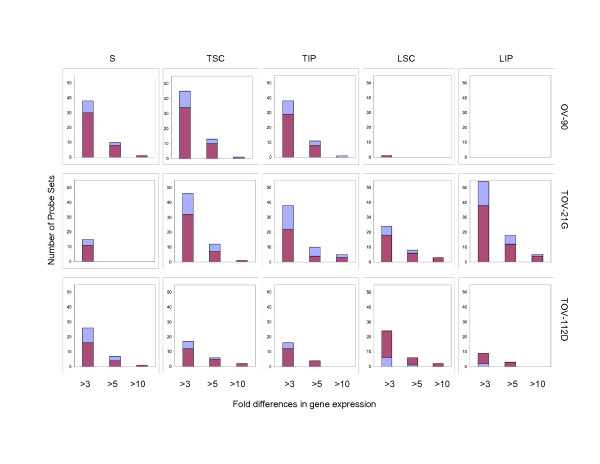
**Differential pattern of gene expression of EOC cell lines grown in different conditions**. The number of differentially expressed genes greater than 3-fold, 5-fold or 10-fold for each alternative growth conditions (spheroid cultures (S), xenograft tumors derived from subcutaneous (TSC) or intraperitoneal (TIP) injection sites in nude mice, and monolayer cultures derived from these tumors (LSC and LIP) relative the monolayer cultures for each EOC cell line (OV-90, TOV-21G and TOV-112D) is shown. The lighter and darker bars represent probe sets that map to the chromosome the 3p and 3q arms, respectively.

To further characterize the gene expression profiles and identify the genes that may be influenced by the growth conditions, we applied a two-way fold-difference comparative analysis approach. As monolayer cultures are often used in molecular genetic assays of cancer derived cell lines, we compared gene expression values of the EOC cell lines grown as monolayer cultures with each of the alternative growth conditions comprised of spheroid cultures (S), nude mouse xenografts at subcutaneous (TSC) or intraperitoneal (TIP) sites, and monolayer cultures of subcutaneous (LSC) and intraperitoneal (LIP) xenografts. We performed two-way comparison analysis based on fold-differences using the expression values which contained at least one high reliability score (or P call) per probe set for each EOC cell line. Using this criterion, the expression values of 692 (60.3%), 739 (64.4%), and 693 (60.4%) probe sets from the total of 1147 chromosome 3 probe sets for OV-90, TOV-21G and TOV-112D, respectively, were evaluated for fold-differences (Table 1). Overall less than 15% of the probe sets exhibited greater than 3-fold differences in gene expression when monolayer cultures were compared with that of any growth condition within each EOC cell line group (Table 1). However, it is apparent that the majority of differences in these comparative analyses occurred within the 3- to 5-fold range, and progressively fewer genes exhibit differences in expression greater than 5-fold and 10-fold (Figure 4). Notable is the strikingly few examples of genes exhibiting at least a 3-fold difference in gene expression in two-way comparisons of monolayer cultures of OV-90 with monolayer cultures of tumors derived from subcutaneous (LSC) or intraperitoneal (LIP) injection sites (Figure 4). This data is consistent with the hierarchical cluster analysis of OV-90 normalized chromosome 3 gene expression data (Figure 1). The fewest differences overall (at 6.6%) were observed in all two-way comparisons of the TOV-112D monolayer culture and any of the alternate growth conditions (Table 1). These results are also consistent with the hierarchical cluster analysis of TOV-112D normalized chromosome 3 gene expression data where overall this EOC cell line exhibited the highest degree of similarity of gene expression (~96.5%) as compared with OV-90 (~92%) (Figure 1) and TOV-21G (92.5%) (Figure 2).

**Table 1:** Two-way comparisons of gene expression values of any alternative growth condition compared with the monolayer cultures.

EOC cell line	Chromosomal location of probe sets	Number of probe sets analyzed	Number (%) of probe sets exhibiting > 3-fold differences in gene expression values in two-way comparisons
OV-90	3p	294	18 (6.1)
OV-90	3q	398	56 (14.1)
OV-90	3	692	74 (10.7)
TOV-21G	3p	339	34 (10.0)
TOV-21G	3q	400	72 (18.0)
TOV-21G	3	739	106 (14.3)
TOV-112D	3p	328	15 (4.6)
TOV-112D	3q	365	31 (8.5)
TOV-112D	3	693	46 (6.6)

### Two-way comparative analysis of the range of gene expression

**Figure 5:**
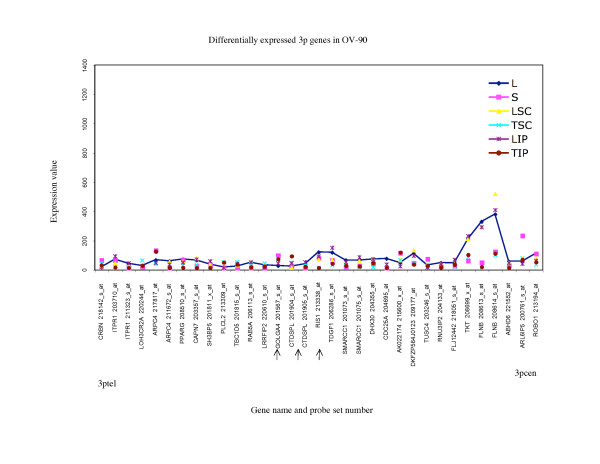
**Range of expression of differentially expressed 3p genes for OV-90**. The expression values of all of the growth conditions are shown for genes exhibiting greater than 3-fold differences in gene expression between the monolayer cultures and any alternative growth condition for OV-90; and greater than 3-fold differences between the maximum and minimum value of expression determined for any growth condition for OV-90. The growth conditions are abbreviated as follows: monolayer culture (L), and alternative growth conditions consisting of spheroid cultures (S), xenograft tumors derived from subcutaneous (TSC) or intraperitoneal (TIP) injection sites in nude mice, and monolayer cultures derived from these tumors (LSC and LIP). For comparative purposes the expression values of the monolayer cultures are linked with a line. The gene name and probe set number are indicated, and are ordered (not to scale) based on the Human Genome Browser March 2006 (hg 18) assembly (UCSC Genome Bioinformatics database). The expression profiles are organized relative to the position of the probe sets (genes) for the 3ptel – 3pcen chromosome arm. The arrows indicate the genes exhibiting differential expression greater than 3-fold in any comparative analyses that were found in common with all three EOC cell lines.

**Figure 6:**
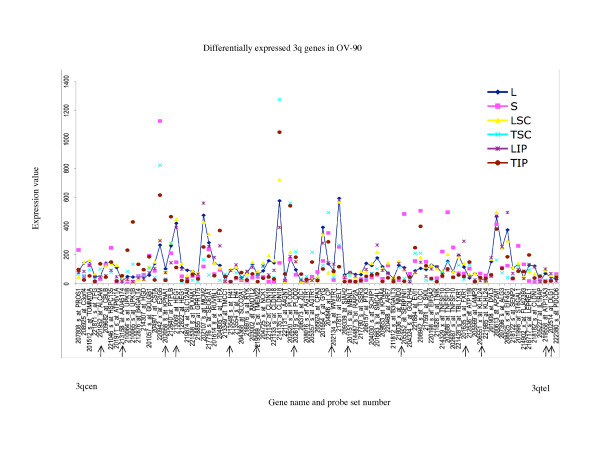
**Range of expression of differentially expressed 3q genes for OV-90**. The expression values of all of the growth conditions are shown for genes exhibiting greater than 3-fold differences in gene expression between the monolayer cultures and any alternative growth condition of OV-90; and greater than 3-fold differences between the maximum and minimum value of expression determined for any growth condition for each OV-90. The growth conditions are abbreviated as follows: monolayer culture (L), and alternative growth conditions consisting of spheroid cultures (S), xenograft tumors derived from subcutaneous (TSC) or intraperitoneal (TIP) injection sites in nude mice, and monolayer cultures derived from these tumors (LSC and LIP). For comparative purposes the expression values of the monolayer cultures are linked with a line. The gene name and probe set number are indicated, and are ordered (not to scale) based on the Human Genome Browser March 2006 (hg 18) assembly (UCSC Genome Bioinformatics database). The expression profiles are organized relative to the position of the probe sets (genes) for the 3qcen – 3qtel chromosome arm. The arrows indicate the genes exhibiting differential expression greater than 3-fold in any comparative analyses that were found in common with all three EOC cell lines.

**Figure 7:**
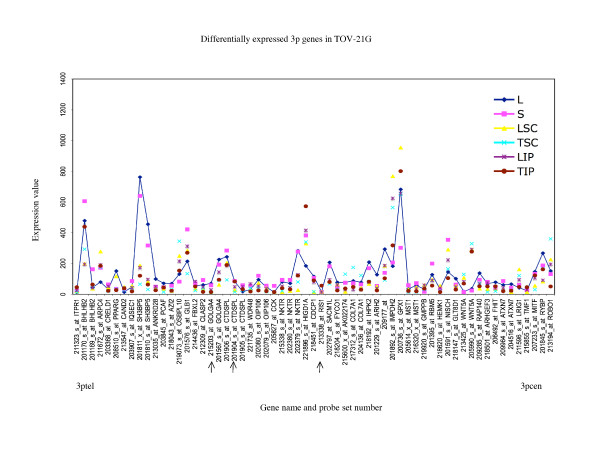
**Range of expression of differentially expressed 3p genes for TOV-21G**. The expression values of all of the growth conditions are shown for genes exhibiting greater than 3-fold differences in gene expression between the monolayer cultures and any alternative growth condition of TOV-21G; and greater than 3-fold differences between the maximum and minimum value of expression determined for any growth condition for TOV-21G. The growth conditions are abbreviated as follows: monolayer culture (L), and alternative growth conditions consisting of spheroid cultures (S), xenograft tumors derived from subcutaneous (TSC) or intraperitoneal (TIP) injection sites in nude mice, and monolayer cultures derived from these tumors (LSC and LIP). For comparative purposes the expression values of the monolayer cultures are linked with a line. The gene name and probe set number are indicated, and are ordered (not to scale) based on the Human Genome Browser March 2006 (hg 18) assembly (UCSC Genome Bioinformatics database). The expression profiles are organized relative to the position of the probe sets (genes) for the 3ptel – 3pcen chromosome arm for TOV-21G. The arrows indicate the genes exhibiting differential expression greater than 3-fold in any comparative analyses that were found in common with all three EOC cell lines.

**Figure 8:**
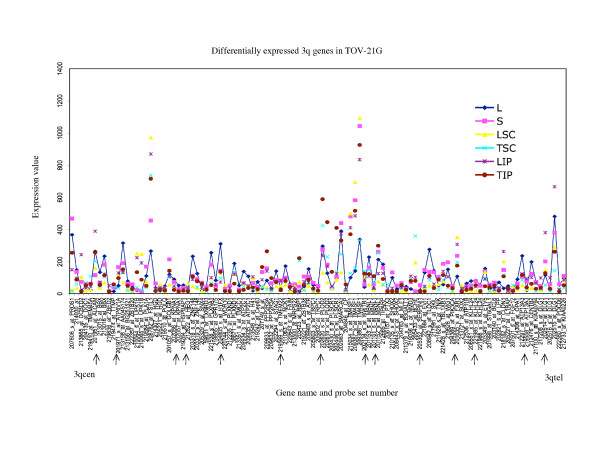
**Range of expression of differentially expressed 3q genes for TOV-21G**. The expression values of all of the growth conditions are shown for genes exhibiting greater than 3-fold differences in gene expression between the monolayer cultures and any alternative growth condition of TOV-21G; and greater than 3-fold differences between the maximum and minimum value of expression determined for any growth condition for TOV-21G. The growth conditions are abbreviated as follows: monolayer culture (L), and alternative growth conditions consisting of spheroid cultures (S), xenograft tumors derived from subcutaneous (TSC) or intraperitoneal (TIP) injection sites in nude mice, and monolayer cultures derived from these tumors (LSC and LIP). For comparative purposes the expression values of the monolayer cultures are linked with a line. The gene name and probe set number are indicated, and are ordered (not to scale) based on the Human Genome Browser March 2006 (hg 18) assembly (UCSC Genome Bioinformatics database). The expression profiles are organized relative to the position of the probe sets (genes) for the 3qcen – 3qtel chromosome arm TOV-21G. The arrows indicate the genes exhibiting differential expression greater than 3-fold in any comparative analyses that were found in common with all three EOC cell lines.

**Figure 9:**
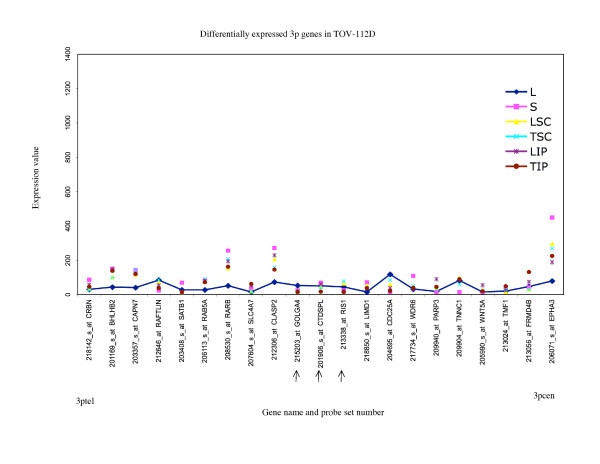
**Range of expression of differentially expressed 3p genes for TOV-112D**. The expression values of all of the growth conditions are shown for genes exhibiting greater than 3-fold differences in gene expression between the monolayer cultures and any alternative growth condition for TOV-112D; and greater than 3-fold differences between the maximum and minimum value of expression determined for any growth condition for TOV-112D. The growth conditions are abbreviated as follows: monolayer culture (L), and alternative growth conditions consisting of spheroid cultures (S), xenograft tumors derived from subcutaneous (TSC) or intraperitoneal (TIP) injection sites in nude mice, and monolayer cultures derived from these tumors (LSC and LIP). For comparative purposes the expression values of the monolayer cultures are linked with a line. The gene name and probe set number are indicated, and are ordered (not to scale) based on the Human Genome Browser March 2006 (hg 18) assembly (UCSC Genome Bioinformatics database). The expression profiles are organized relative to the position of the probe sets (genes) for the 3ptel – 3pcen chromosome arm for TOV-112D. The arrows indicate the genes exhibiting differential expression greater than 3-fold in any comparative analyses that were found in common with all three EOC cell lines.

**Figure 10:**
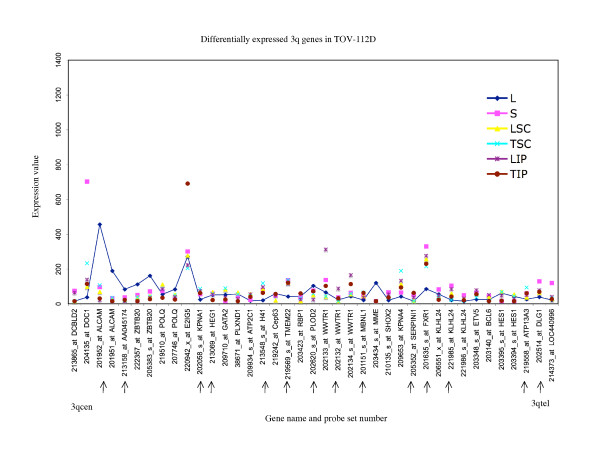
**Range of expression of differentially expressed 3q genes for TOV-112D**. The expression values of all of the growth conditions are shown for genes exhibiting greater than 3-fold differences in gene expression between the monolayer cultures and any alternative growth condition for TOV112D; and greater than 3-fold differences between the maximum and minimum value of expression determined for any growth condition for TOV-112D. The growth conditions are abbreviated as follows: monolayer culture (L), and alternative growth conditions consisting of spheroid cultures (S), xenograft tumors derived from subcutaneous (TSC) or intraperitoneal (TIP) injection sites in nude mice, and monolayer cultures derived from these tumors (LSC and LIP). For comparative purposes the expression values of the monolayer cultures are linked with a line. The gene name and probe set number are indicated, and are ordered (not to scale) based on the Human Genome Browser March 2006 (hg 18) assembly (UCSC Genome Bioinformatics database). The expression profiles are organized relative to the position of the probe sets (genes) for the 3qcen – 3qtel chromosome arm for TOV-112D. The arrows indicate the genes exhibiting differential expression greater than 3-fold in any comparative analyses that were found in common with all three EOC cell lines.

To further characterize the differences in the gene expression patterns, we examined the range (maximum and minimum) of the expression values exhibited by all alternative growth conditions. Two-way comparative analysis was performed between the maximum and minimum values of expression observed for probe sets for each EOC cell line group. A minimum 3-fold cut-off was used to characterize differences in gene expression. Overall less than 23% of probe sets exhibited greater than 3-fold differences in gene expression in this comparative analysis in any EOC cell line group (Table 2). It is apparent in this analysis that the majority of differences between the maximum and minimum values of gene expression occurred within the 3- to 5-fold range, and progressively fewer genes exhibit differences in the range greater than 5- to 10-fold or greater than 10-fold. These observations are evident when gene expression values for probe sets exhibiting at least a 3-fold difference in the range of expression for any growth condition are shown graphically as in Figures 5 to 10. For example only 10 of 122 (8%) probe sets representing eight genes, *FLNB *on the 3p arm (Figure 5) and *UPK1B, H1FX, CLDN18, AGTR1, EIF4G1, SERPINI1*, and *LEPREL1 *on the 3q arm (Figure 6), exhibited greater than 10-fold differences in gene expression in the analysis of OV-90.

**Table 2:** Two-way comparisons of the maximum and minimum value of expression exhibited by a probe set of any alternative growth condition

		Number (%) of probe sets exhibiting > 3-fold differences in gene expression values in two-way comparisons			
		
EOC cell line	Number of probe sets analyzed	> 3-fold	> 3-to-5-fold	> 5-to-10-fold	> 10-fold
OV-90	692	122 (17.6)	81 (11.7)	31 (4.5)	10 (1.4)
TOV-21G	739	168 (22.7)	131 (17.7)	27 (3.7)	10 (1.4)
TOV-112D	693	60 (8.7)	43 (6.2)	14 (2.0)	3 (0.4)

There were 17 genes which were found differentially expressed greater than 3-fold in all EOC cell lines (Additional file 1). These genes may represent those that could be affected by growth condition or tumor microenvironment [13]. Notable is that the patterns of expression of these 17 genes were not necessarily the same for each growth condition when one cell line is compared with another. For example in OV-90, the maximum value of expression of *RIS1 *was found with the monolayer culture (L) and both subcutaneous (TSC) and intraperitoneal (TIP) xenografts exhibited the lowest values of expression of this gene (Figure 5), whereas the highest level of expression of *RIS1 *in TOV-112D was found with the subcutaneous (TSC) xenograft sample and the lowest value was observed with intraperitoneal (TIP) xenograft (Figure 9).

## Discussion

In this study, we described chromosome 3 transcriptome changes for three well characterized EOC cell lines (OV-90, TOV-112D, and TOV-21G) that each responded differently in relation to various growth conditions such as in three dimensional spheroid culture and nude mouse xenograft models, relative to the conventional monolayer culture. However, the alternative *in vitro *and *in vivo *growth conditions of tumorigenic EOC cell lines appeared to have modestly influenced the expression of chromosome 3 genes. This was reflected in the hierarchical cluster analysis where there was an overall high degree of correlation (> 90%) in gene expression in each EOC cell line group tested irrespective of growth condition. It was also reflected in the two-way comparative analyses where a 3-fold cut-off was applied. Although we have previously shown that replicates of Affymetrix GeneChip^® ^expression data derived from the EOC cell lines grown as monolayer cultures were highly reproducible [5,6], a lower cut-off (such as a 2-fold cut-off) would have captured differences in gene expression attributable to experimental variability [5,17]. Unlike earlier studies using Affymetrix GeneChip^® ^expression microarrays of the EOC cell lines, we have used a lower threshold level of 15 rather than 50 or 100 depending on the GeneChip^® ^used [5,6,8-10]. A lower threshold value would increase the number of differentially expressed genes occurring in the low range of gene expression and this perhaps explains the large number of differentially expressed genes with values falling below 150 for all growth conditions (see Figures 5 to 10). The two-way comparison analyses were consistent with hierarchical cluster analyses which indicated a high correlation in gene expression patterns in the EOC cell line regardless of growth condition. Our results with chromosome 3 genes were consistent with whole genome transcriptome analyses of the EOC cell lines which also showed a high correlation (> 85%) of gene expression regardless of growth condition suggesting that microenvironment modestly influenced gene expression [13].

The EOC cell line lines exhibited unique patterns of gene expression as shown by the hierarchical cluster analysis. These unique differences are also reflected in a previous global analyses of gene expression from the entire Affymetrix U133A microarray [13]. Thus while gene expression profiles of OV-90 cell line grown as tumors or spheroid clustered together, which may indicate that gene expression patterns could be associated with growth as 3D structures, this was not the case in chromosome 3 transcriptome profiles for TOV-21G and TOV-112D. The differences in the clustering patterns and differentially expressed genes observed in the three EOC cell lines was not surprising. The EOC cell lines were derived from long-term passages of tumor tissues representing different histopathological subtypes of ovarian cancer [2]. These EOC cell lines also differ in their molecular genetic characteristics, in that OV-90 and TOV-112D harbor somatic mutations in *TP53*, whereas TOV-21G harbors a somatic mutation in *KRAS *and exhibits microsatellite instability. OV-90 also is monoallelic for the 3p arm, however this gross genomic anomaly did not significantly impact on global patterns of gene expression of the chromosome 3p arm as assayed by Affymetrix expression analyses of this cell line and the other EOC cell line used in the present study [3,8,15]. The EOC cell lines also differ in their response to ionizing radiation and chemotherapeutic agents [3]. Thus the unique patterns of gene expression as shown in Figures 5 to 10 could in part reflect molecular genetic differences of the these cell lines.

Given the molecular genetic differences in the EOC cell lines, it is not surprising that there were few similarly differentially expressed genes found in common with all of EOC cell lines. Indeed there were only 17 genes in common in all three EOC cell lines which exhibited differential expression greater than 3-fold in all comparative analyses (Additional file 1). A review of gene ontology suggests that some examples of the differentially expressed genes have been associated with cellular shape (*ARPC4 *and *NCK1*), cell growth and division (*CDC25A *and *SKIL*), and extracellular signaling/cell-cell junctions (*ROBO1, SKIL, TM4SF1 *and *WNT5A*) (Additional file 1). Some of these genes have recently been identified as differentially expressed in ovarian cancer samples relative to normal tissue. For example, *TMEM158, HEG1, PLOD2 *and *ATP13A3*, were recently found differentially expressed greater than 3-fold in a comparative analysis of primary cultures of normal ovarian surface epithelial cells and malignant serous ovarian tumors [7]. However, the 17 differentially expressed genes observed in common with all three EOC cell lines do not necessarily exhibit that the same differences in gene expression patterns relative to monolayer cultures, suggesting that they each may have responded differently to alternative growth conditions. Further analysis is required to determine if these 17 genes are indeed responding to differences in microenvironment as consequence of growth alternative growth conditions.

Future experiments are required to determine if the differences observed in the EOC cell lines grown in alternative conditions are biologically relevant or a reflection of experimental design. The magnitude of the differences in gene expression observed in the EOC cell lines grown under the various *in vitro *and *in vivo *growth conditions may all still be significantly different when each is compared with normal cells [7]. The EOC cell lines, with their capacity to grow in different contexts, provides an opportunity to further examine the biological relevance of transcriptional differences that may be influenced by the microenvironment wherein which they are propagated. Recently our group has applied such a strategy to specifically identify genes transcriptionally modified based on microenvironment, and one such gene, *S100A6*, was found differentially expressed relative to culture conditions and further validated by RT-PCR and immunohistochemistry [13]. While this finding may be disconcerting and discourage the use of *in vitro *model systems for studying gene candidates, our results in the present study show that a high correlation of gene expression in the transcriptomes generated from ovarian cancer cell lines propagated in different contexts. Overall these results attests to the validity of the EOC cell lines as an *in vitro *model for studying gene candidates but point out that some genes may be influenced by microenvironment, a factor that should be taken into consideration when investigating the molecular biology of specific genes. As our EOC cell lines are amenable to propagation in alternative growth conditions one could assay and further investigate the magnitude of transcriptional effects for specific candidate genes of interest and their consequences at the protein level to further understand the biological relevance gene expression differences associated with microenvironment.

## Conclusion

The ability to culture tumorigenic EOC cells under different *in vivo *and *in vitro *growth conditions affords the opportunity to study gene expression of candidates in contexts that more closely mimic tumor growth *in vitro*. However, the analyses of chromosome 3 transcriptomes are highly comparable within each EOC cell line context. These observations would argue that gene expression studies using monolayer cultures of ovarian cancer lines is still a viable option for initial studies involving the characterization of gene expression pattern of candidate genes.

## Competing interests

The authors declare that they have no competing interests.

## Authors' contributions

NALC participated in the study design of expression analysis, performed the data analysis, and drafted the manuscript. MZ performed experiments involving the growth of the EOC cell lines in different conditions. DMP participated in the study design. A-MM-M supervised the growth conditions assays and participated in the study design. PNT conceived the chromosome 3 study and study design, and drafted the manuscript. All authors have read the manuscript and approved the final manuscript.

## Pre-publication history

The pre-publication history for this paper can be accessed here:



## Supplementary Material

Additional file 1 Table S1: Genes exhibiting greater than 3-fold difference in gene expression in common in all EOC cell lines. Click here for file
